# Phenotype overlap in glial cell populations: astroglia, oligodendroglia and NG-2(+) cells

**DOI:** 10.3389/fnana.2015.00049

**Published:** 2015-05-12

**Authors:** Badrah Alghamdi, Robert Fern

**Affiliations:** ^1^Department of Cell Physiology and Pharmacology, University of LeicesterLeicester, UK; ^2^Peninsula School of Medicine and Dentistry, University of PlymouthPlymouth, UK

**Keywords:** astrocyte, development, glia, oligodendrocyte, NG-2

## Abstract

The extent to which NG-2(+) cells form a distinct population separate from astrocytes is central to understanding whether this important cell class is wholly an oligodendrocyte precursor cell (OPC) or has additional functions akin to those classically ascribed to astrocytes. Early immuno-staining studies indicate that NG-2(+) cells do not express the astrocyte marker GFAP, but orthogonal reconstructions of double-labeled confocal image stacks here reveal a significant degree of co-expression in individual cells within post-natal day 10 (P10) and adult rat optic nerve (RON) and rat cortex. Extensive scanning of various antibody/fixation/embedding approaches identified a protocol for selective post-embedded immuno-gold labeling. This first ultrastructural characterization of identified NG-2(+) cells revealed populations of both OPCs and astrocytes in P10 RON. NG-2(+) astrocytes had classic features including the presence of glial filaments but low levels of glial filament expression were also found in OPCs and myelinating oligodendrocytes. P0 RONs contained few OPCs but positively identified astrocytes were observed to ensheath pre-myelinated axons in a fashion previously described as a definitive marker of the oligodendrocyte lineage. Astrocyte ensheathment was also apparent in P10 RONs, was absent from developing nodes of Ranvier and was never associated with compact myelin. Astrocyte processes were also shown to encapsulate some oligodendrocyte somata. The data indicate that common criteria for delineating astrocytes and oligodendroglia are insufficiently robust and that astrocyte features ascribed to OPCs may arise from misidentification.

## Introduction

Glial cells expressing the NG-2 proteoglycan act as oligodendrocyte precursor cells (OPCs) and are retained in the mature CNS where they form a reservoir of progenitors that may be significant for the development of effective treatment strategies for common neurological diseases (Nishiyama et al., 2009). NG-2(+) cells can receive synaptic input and therefore they challenge the dogmatic separation of neural cells into either neuronal/excitable or glial/support roles. Historically, cells with features of NG-2(+) cells have been variously described as “small glioblasts” (Vaughn, 1969), “3rd glial element” cells (Vaughn and Peters, 1968), “β-astrocytes” (Reyners et al., 1982),“oligodendrocyte/type 2 astrocytes” (O2A) (Raff et al., 1983), “smooth protoplasmic astrocytes” (Levine and Card, 1987), “OPCs” (Ong and Levine, 1999), “astons” (Matthias et al., 2003), “polydendrocytes” (Nishiyama et al., 2009) and “synantocytes” (Leoni et al., 2009). This diversity of nomenclature reflects the degree of uncertainty regarding the cells' ontogeny and their physiological functions. In particular, early studies noted the morphological similarity between NG-2(+) cells and astrocytes, while subsequent reports have documented astrocyte-type features such as process extension into the node of Ranvier, synapses encapsulation (Levine and Card, 1987; Ong and Levine, 1999; Wigley and Butt, 2009), and reactive gliosis with features of astrocytosis (Greenwood and Butt, 2003; Lytle et al., 2009). The degree to which these cells may exhibit astrocyte-type behavior remains controversial, as does the extent to which astrocytes and NG-2(+) cells share a common cell fate.

## Materials and methods

UK home office regulations were followed for all experimental work which was conducted in accordance with the Council Directive 2010/63EU of the European Parliament and the Council of 22 September 2010 on the protection of animals used for scientific purposes. The animal welfare and ethics committee of University of Leicester approved all the experimental protocols. Rat optic nerves (RONs) were dissected from Lister-hooded rats on post-natal day 0 (referred to as “P0”), P8-12 (“P10”), or >P80 (adult).

### Immuno-histochemistry

RONs were lightly fixed in 2% paraformaldehyde/0.1M PBS for 30 min prior to incubation in 0.1M PBS plus 20% sucrose w/v for 5 min and freeze-sectioning. Twenty micro meter sections were subsequently blocked for 60 min in 0.1M PBS +10% normal goat serum +0.5% Triton-X 100 (PBST). Sections were then incubated in this solution (plus primary antibody) overnight at 4°C. Antibodies were raised against NG-2 (1:100 Millipore MAB5384 and GFAP 1:200 Sigma G4546). Staining was detected using appropriate Alexa-conjugated secondary antibodies (1:1000 Molecular probes). Primary antibody omission controls were blank. Images were collected using an Olympus confocal microscope and image stacks were analyzed using NIH Image-J.

### Immuno-electron microscopy (I-EM)

After extensive testing using a variety of fixation protocols (3% glutaraldehyde in Sorensen's +1% osmium / 3% glutaraldehyde +2% paraformaldehyde in Sorensen's +1% osmium), embedding (propylene oxide + Spurr's resin / propylene oxide + Agar low viscosity resin / ethanol + LR White resin), etching and staining protocols, the following technique produced acceptable results: RONs were dissected in Sorenson's buffer, washed in 0.1 M sodium cacodylate buffer (2 mM CaCl_2_/pH 7.4) and post-fixed in 2% formaldehyde +3% glutaraldehyde in cacodylate buffer overnight. Nerves were then washed prior to secondary fixation (1% osmium tetroxide / 1.5% potassium ferricyanide) and washed again prior to tertiary fixation (2% uranyl acetate). RONs were sequentially dehydrated, washed in propylene oxide, and embedded using the following steps: (a) 2:1 propylene oxide + modified Spurr's low viscosity resin for 90 min; (b) 1:1 propylene oxide + modified Spurr's resin for 60 min; (c) 1:2 propylene oxide + modified Spurr's resin for 60 min; (d) 100% Spurr's low viscosity resin (30 min, then overnight, then 180 min); and (e) polymerization for 16 h at 60°C.

Post-embedded I-EM for NG-2 has not previously been achieved. We tested five different antibodies and antibody cocktails (4 h primary staining followed by washing and secondary staining for 60 min) in sections that had been either etched (saturated sodium metaperiodate 30 min), blocked (PBST 30 min), etched and blocked, or left untreated. The primary antibodies tested were: (a) Rabbit polyclonal AB62341 from Abcam; (b) Rabbit polyclonal generously gifted from William Stallcup; (c) Mouse monoclonal MAB5384 from Millipore; (d) Mouse monoclonal cocktail generously gifted from William Stallcup; and (e) Mouse monoclonal cocktail (D120.43/D4.11/N143.8/N109.6 clones) 37-2700 from Zymed. Staining using all five antibodies/cocktails was tested on ultrathin sections at 1:200, 1:100, 1:50 and 1:20 and reactivity detected using a goat anti-mouse or goat anti-rabbit 30 nm gold conjugate, as appropriate (1:50; Sigma). Ultrathin sections were counter-stained with uranyl acetate and lead citrate and examined with a Jeol 100CX electron microscope.

## Results

**Figure 1:**
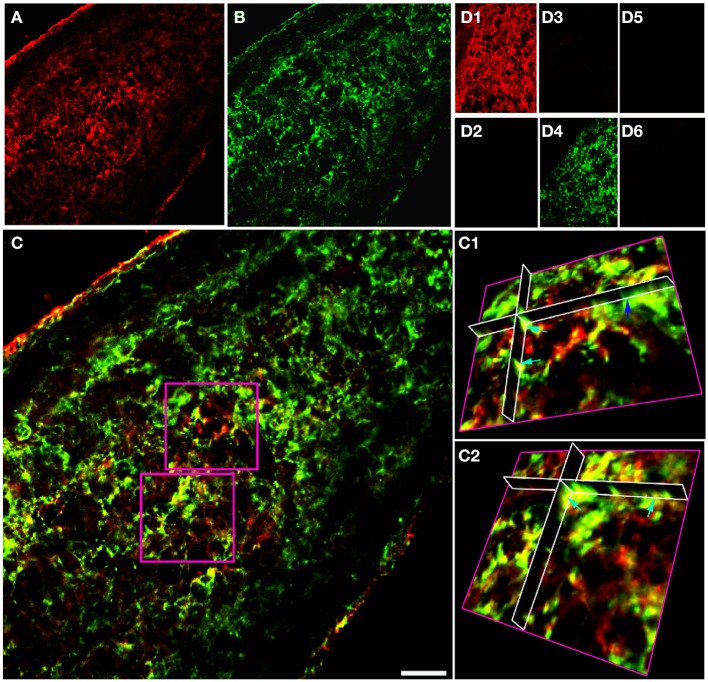
**GFAP and NG-2 co-localization in P10 RON glial cells. (A)** GFAP immuno-reactivity (red). **(B)** NG-2 immuno-reactivity (green). **(C)** Overlay with boxed areas shown at higher gain as 3-D projections in **(C1)** and **(C2)**. Note the NG-2 co-localization (red or orange regions e.g., light blue arrows) in parts of cells that may also have regions that are only GFAP(+) (green regions e.g., dark blue arrow). **(D)** Controls showing GFAP staining **(D1)** and absence of NG-2 staining **(D2)** when the NG-2 antibody was omitted from the otherwise identical protocol; NG-2 staining **(D3)** and no GFAP staining **(D4)** when the GFAP antibody was omitted, and the absence of any staining when both primary antibodies were omitted (**D5,D6**). All images were collected and displayed using identical settings. Bar = 10 μm.

Double immuno-labeled P10 RON 10 μm projections indicate apparent regions of single and co-expression for GFAP/NG-2 (Figures 1A–C). Ortho-projections revealed non-overlapping regions of expression in individual cells (e.g., the dark blue arrow in Figure 1C1 shows an NG-2(+)/GFAP(−) soma) and regions of co-expression in both cell somata and processes (e.g., Figures 1C1,C2, light blue arrows). The analysis indicates a large population of cells express both proteins, but often in separate structures. Cell counting of stack projections in P 10 RON showed 38.5 +/− 2.7% of cells were GFAP(+)/NG-2(−), 28.5 +/− 2.6% were GFAP(−)/NG-2(+) and 33.0 +/− 5.3% were GFAP(+)/NG-2(+) (544 cells analyzed from 5 sections). Control staining with omission of one or both primary antibodies showed no cross-labeling of secondary antibodies, bleed through of channels, significant background fluorescence, or non-specific staining (Figures 1D1–D6). All comparable images were collected using identical image and acquisition settings to allow direct comparison of test and controls.

**Figure 2:**
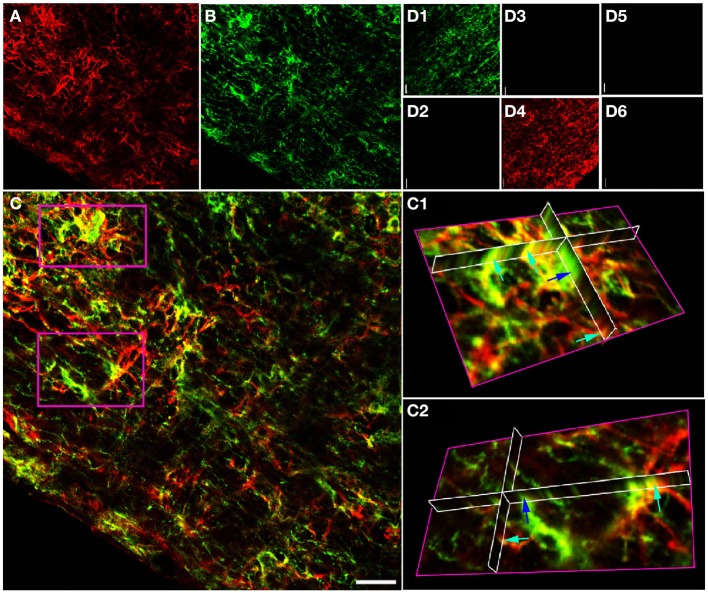
**GFAP and NG-2 co-localization in the adult RON. (A)** GFAP immuno-reactivity (red). **(B)** NG-2 immuno-reactivity (green). **(C)** Overlay with boxed areas shown at higher gain as 3-D projections in **(C1)** and **(C2)**. Note the NG-2 co-localization (red or orange, e.g., light blue arrows) in parts of cells that may express GFAP alone in other regions, and in cells that are only GFAP(+) (e.g., dark blue arrow). **(D)** Controls showing NG-2 staining **(D1)** and absence of GFAP staining **(D2)** when the GFAP antibody was omitted from the otherwise identical protocol; GFAP staining **(D4)** and no NG-2 staining **(D3)** when the NG-2 antibody was omitted, and the absence of any staining when both primary antibodies were omitted (**D5,D6**). All images were collected and displayed using identical settings. Bar = 10 μm.

Projections of GFAP and NG-2 immuno-staining in adult RON (Figures 2A–D) show a mixture of single and double labeling of cells and processes. Ortho-projections of the boxed areas (Figures 2C1,C2) again revealed examples of single (e.g., dark blue arrows) and double (e.g., light blue arrows) staining. Cell counting of stack projections showed 19.5 +/− 1.4% of cells were GFAP(+)/NG-2(−), 20.6 +/− 2.6% were GFAP(−)/NG-2(+) and 59.9 +/−1.6% were GFAP(+)/NG-2(+) (369 cells analyzed from 5 sections). This was a higher apparent proportion of co-expressing cells than found in the P10 nerves.

**Figure 3:**
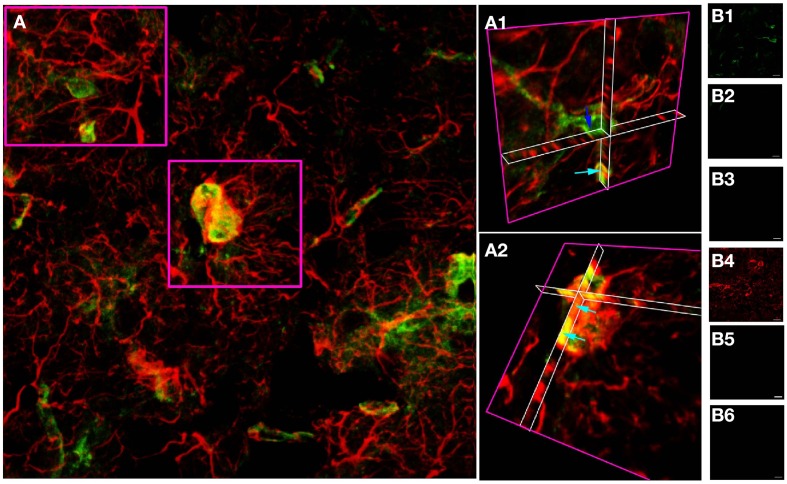
**GFAP and NG-2 co-localization in adult cortical gray matter. (A)** GFAP immuno-reactivity (red) and NG-2 immuno-reactivity (green), with the two boxed areas shown at higher gain as 3-D projections in **(A1)** and **(A2)**. Note the NG-2 co-localization (e.g., light blue arrows) in parts of cells that may also have regions that are only GFAP(+), while other NG-2(+) cells are GFAP(–) (e.g., dark blue arrow). **(B)** Controls showing GFAP staining **(B1)** and absence of NG-2 staining **(B2)** when the NG-2 antibody was omitted from the otherwise identical protocol; NG-2 staining **(B4)** and no GFAP staining **(B3)** when the GFAP antibody was omitted, and the absence of any staining when both primary antibodies were omitted (**B5,B6**). All images were collected and displayed using identical settings. Bar = 10 μm.

A similar approach in adult cortical gray matter produces comparable co-stained cells (Figure 3), with a population of NG-2(+) cells having no apparent regions of GFAP expression (Figure 3A1, dark blue arrow) and a population that co-expresses both proteins (Figure 3A2, single blue arrows). Similar data were observed in the adjacent sub-cortical white matter structures. Cell counting of stack projections showed 68.9 +/− 3.8% of cells were GFAP(+)/NG-2(−), 10.8 +/−2.8% were GFAP(−)/NG-2(+) and 20.3 +/−1.4% were GFAP(+)/NG-2(+) (384 cells analyzed from 5 sections) in the gray matter. As in the examples shown, it was often the case in cortical sections that GFAP reactivity was localized individually to the processes of cells that expressed NG-2 on the soma.

NG-2(+) cells and GFAP(+) cells may form close morphological arrangements that are problematic to sufficiently resolved using the immuno-fluorescent approach described above. We therefore developed an immuno-gold post-embedded methodology for the ultrastructural analysis of NG-2(+) cells in P10 RON. Retaining antigenicity for NG-2 in tissue prepared for I-EM proved difficult. A variety of polyclonal and monoclonal antibodies (and cocktails) were tested over a range of concentrations on P10 RONs collected under a number of fixation and embedding protocols (see Materials and Methods). Cell-specific staining was achieved with a polyclonal antibody on nerves using an overnight primary fixation (3% glutaraldehyde/2% formaldehyde) in cacodylate buffer followed by two further fixation steps, gradual embedding in modified Spurr's resin, and sodium metaperiodate etching. Using this protocol, staining levels were low but selective. Blinded counting of gold particles in 10 grid sections each from three independent nerves found 7.4% in axons (*n* = 11/1239 axons), 59.4% in glia somata (*n* = 35/101 somata), 27.9% in glial processes and 6.1% in glial nuclei (*n* = 9/101 nuclei; total *n* = 148 particles). 87.3% of staining was therefore in the glial cell membrane or cytoplasm, with the remaining gold particles showing a background level of non-specific staining in axons and nuclei. This level of background staining is consistent with a number of other studies using I-EM in RON (e.g., Alix et al., 2008; Arranz et al., 2008; Alix and Fern, 2009). In total, six fixation and embedding protocols were attempted for all 5 antibody/cocktail mixtures over 4 concentration ranges; only the one successful protocol was identified, with non-selective staining and null-staining proving to be the major shortfalls of the other fixation/embedding staining combinations.

**Figure 4:**
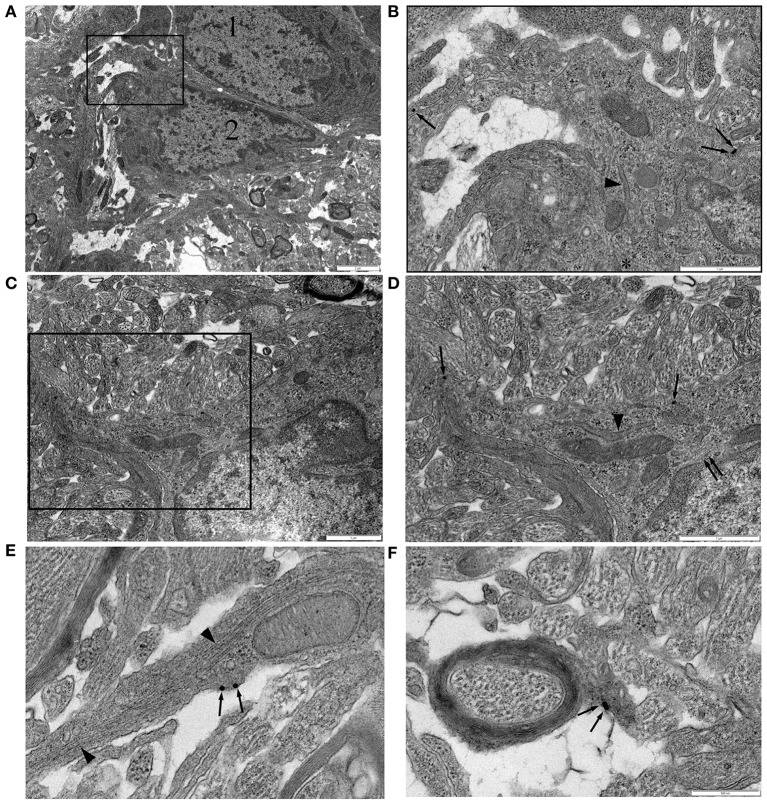
**NG-2 immuno-gold labeling in P10 RON. (A,B)** Two closely apposed glial soma (“1” and “2”). Cell “1” has features typical of an early cell of the oligodendroglial lineage including an ovoid nucleus and narrow bore ER. Cell “2” has features that are typical of astrocytes in this preparation. The boxed area is shown at higher gain in (**B**). Note the gold particles (some indicated by arrows) which identify this cell as NG-2(+). A lobular nuclear morphology with clustered chromatin under the nuclear envelope and a wide bore ER (arrow heads) are astrocyte features. The cytoplasm also contains microtubules (e.g., asterisk). Glial filaments cannot be positively identified in this cell. **(C,D)** Another NG-2(+) cell with astrocyte features which does express glial filaments (double arrows). Boxed area shown at higher gain in **(D)**. **(E)** High-gain micrograph of NG-2 staining in glial processes (arrows) which contains glia filaments (arrowhead). **(F)** An example of NG-2(+) (arrows) oligodendrocyte processes ensheathing an axon.

In P10 RON, gold particles were frequently detected in the cell membrane or cytoplasm of glial somata (Figures 4A–D, single arrows). Labeled cells most frequently had a wide-bore endoplasmic reticulum (ER; Figures 4A–C, arrow heads) and a granular chromatin that was often clustered under the nuclear envelope. These cells occasionally exhibited stacked glial filaments in the cytoplasm (Figures 4A–C, double arrows) and have the classic features of astrocytes, which are the predominant type of cell present in the nerve at this age (Vaughn and Peters, 1967; Vaughn, 1969). NG-2 reactivity (gold particles) was also present in glial processes that did not contain obvious glial filaments and in some that did (Figure 4E), as well as in oligodendrocyte processes that had initiated axon wrapping and myelination (Figure 4F). Staining was rarely observed in undifferentiated glioblasts which will include OPCs, but such cells make up <10% of the glial population at this age (Vaughn, 1969; Barres et al., 1992). The ultrastructural analysis therefore aligns with the confocal immuno-fluorescent data showing NG-2(+) GFAP(+) astrocytes in the neonatal optic nerve.

**Figure 5:**
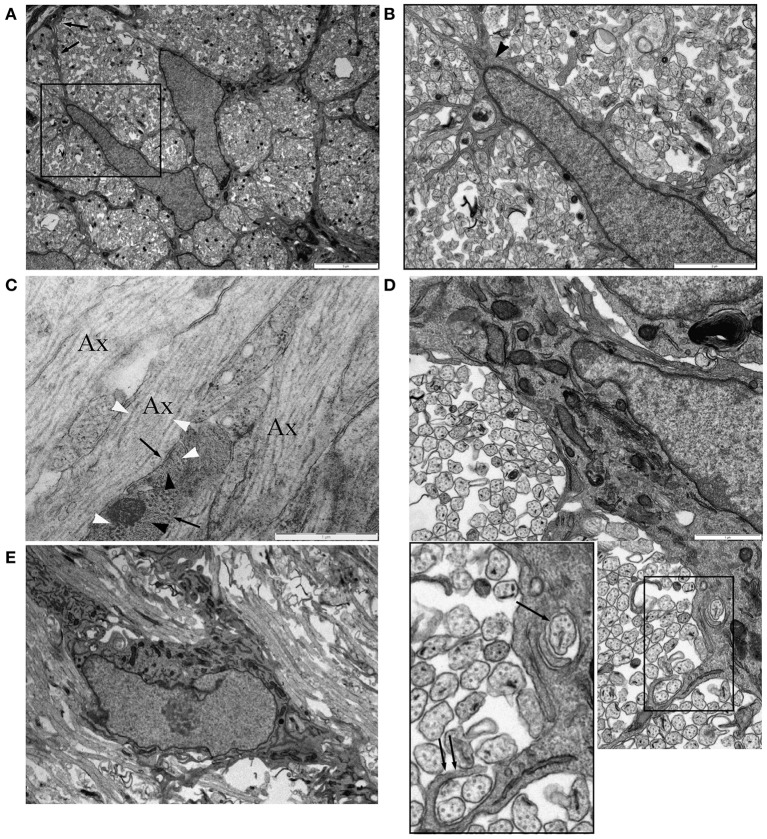
**Astrocytes in P0 RON ensheath axons. (A)** Low magnification micrograph showing two neighboring astrocytes in cross-section RON. The boxed area is shown at higher power in **(B)**, revealing the highly ramified nature of the many fine processes that extend from the soma and main branches of the cell, which can be identified as an astrocyte due to occasional glial filaments (arrow head) and the contribution the cell makes to the glial limitans (**A**: arrows). **(C)** Long-section of P0 RON at high gain, showing the close apposition of pre-myelinated axons (“Ax”) and astrocyte processes (arrows). Microtubules (white arrow heads) and neurofilaments are arranged longitudinally in axons while glial filaments (black arrow heads) are arranged generally transversely in astrocyte processes. **(D)** Montage showing an astrocyte ensheathing a single axon (arrow) and extending a finger process around two axons (double arrow). Boxed area shown at higher gain in the inset. **(E)** An astrocyte shown in long-section extending thick processes parallel to pre-myelinated axons.

Co-expression of the early oligodendroglial lineage marker NG-2 and astrocyte marker GFAP in glial cells of the optic nerve raises questions about how these two cell fates are distinguished. We examined P0 RON, a developmental point before the wide-spread arrival of OPC (Vaughn, 1969; Small et al., 1987; Barres et al., 1992) and a point when astrocyte production has peaked (Vaughn and Peters, 1967; Vaughn, 1969; Skoff et al., 1976; Skoff, 1990). A population of astrocytes can be unambiguously identified at this age, for example by the radiating processes found in cross-sections that contribute to the glial limitans, a wholly astrocytic structure (Figure 5A, arrows). Such cells often expressed small bundles of glial filaments in the somata and processes (Figure 5B, arrow head) and have a wide-bore ER typical of astrocytes, see (Vaughn and Peters, 1967; Vaughn, 1969; Federoff and Vernadakis, 1986) (Figures 5B,D,F). Thick astrocytes processes separate axons into fascicles (Figure 5A) and run parallel to axons along the nerve (Figure 5F). “Finger processes” have previously been described in these cells (Figure 5B) and had been thought to represent process extension prior to fasciculation but these structures exist in cells that appear to have completed fasciculation (Figures 5A,B) and often wrap around pre-myelinated axons of various diameters (Figure 5D). Transverse sections confirm the presence of ensheathing processes (Figure 5C), which sometimes contain glial filaments (Figure 5C, black arrows). Unlike ensheathing oligodendrocyte processes, NG-2 reactivity was not observed on the ensheathing astrocyte processes.

**Figure 6:**
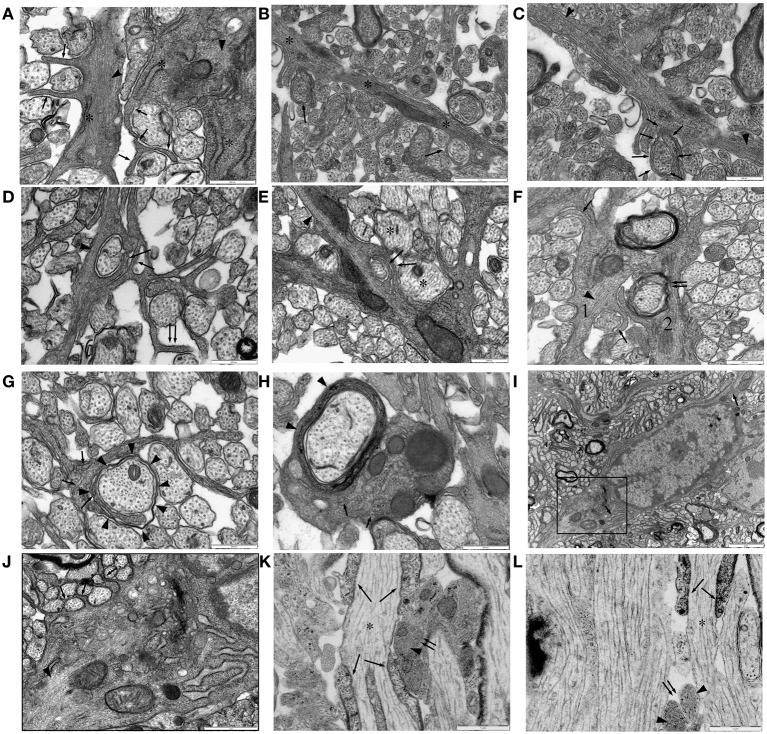
**Ensheathing astrocytes in P10 RON. (A–I) Micrographs of nerve cross-sections. (A)** Two astrocyte processes identified by the presence of glial filaments (dark arrow heads, typically oriented transversely) also containing wide-bore ER (“^*^”) and extending finger-processes between small diameter pre-myelinated axons (arrows). **(B)** An astrocyte processes oriented radially (“^*^”) navigates between pre-myelinated axons. Ensheathing processes either partially or completely surround several neighboring small-diameter axons (arrows). **(C)** The top left section of **(B)** shown at higher gain, reveals extensive glial filaments within the main astrocyte processes (arrow heads) and cytoplasmic continuity with an ensheathing processes (arrows). **(D,E)** Examples of double-wrapping of small diameter pre-myelinated axons (dark arrows) by glial filament-containing (arrow heads) processes. Note the presence of neighboring larger-diameter pre-myelinating axons that are not ensheathed (e.g., “^*^”), and the “en-passent” nature of the ensheathment, with processes continuing on to navigate between neighboring axons (e.g., double-arrows). **(F)** Two glial processes. Process “1” contains glial filaments (dark arrow head) and has partially ensheathed several small axons (e.g., dark arrows). Process “2” appears to have wrapped several layers around a larger axon (double arrows) and looks similar to “1” except that it contains no obvious filaments. **(G)** A glial processes containing neurofilaments, some oriented transversely (arrows) but no glial filaments, navigates between small diameter axons and has initiated wrapping of a large axon (arrow heads). **(H)** Oligodendrocyte processes showing multiple layers of myelin (arrow head) contain neurofilaments (arrows) but no glial filaments. Note the presence of glial filaments in neighboring glial processes. **(I)** Astrocyte somata, note the wide-bore ER (arrows) and the characteristic hetero-chromatin. The boxed area is shown at higher gain in **(J)**. **(J)** Note the glial filaments (arrow head) and the finger processes originating directly from the soma ensheathing small diameter axons (e.g., arrows). **(K,L)** Examples of long-section micrographs showing early myelination of a node of Ranvier in **(K)** and a hemi-node in **(L)** (“^*^”). Oligodendroglial processes navigate along the axon (arrows), and glial filament-filled (arrow heads) astrocyte processes cluster adjacent to the putative nodal membrane but do not wrap around it (double arrows).

Numerous finger processes are also found in P10 RON and are more likely to contain glial filaments than other regions of the astrocyte (Figure 6A). Examples are present that bifurcate to initiate ensheathment of small axons (Figures 6A,B) and double finger processes erupting from large processes to flank axons from either side (Figures 6A,D,E), while single finger processes occasionally wrap the entire axon circumference (Figure 6C). Multiple layers of ensheathment can be seen (Figures 6D,E) but compact myelin cannot be unambiguously associated with these processes (Figure 6F) and myelinating oligodendrocyte processes never contain any of the features of astrocytes (e.g., stacked glial filaments, wide-bore ER), although single glial filaments are apparent on occasion (Figure 6H). Fine processes that lack classical astrocyte features are present and make close connections with larger pre-myelinated axons (Figure 6G) and may be oligodendroglial. Small axons are also sometimes embedded in the surface of astrocyte somata that have prominent glial filament expression and wide-bore ER (Figures 6I,J). In long section, glial filament-containing processes are frequently found in early nodes of Ranvier (Figure 6K) and at hemi-nodes sites where one myelinating process has yet to arrive (Figure 6L), but were not observed to wrap around the larger myelinating axon at these points. If axons wrapping by astrocytes is a feature of early node of Ranvier sites, it must, therefore, reverse as myelination progresses.

**Figure 7:**
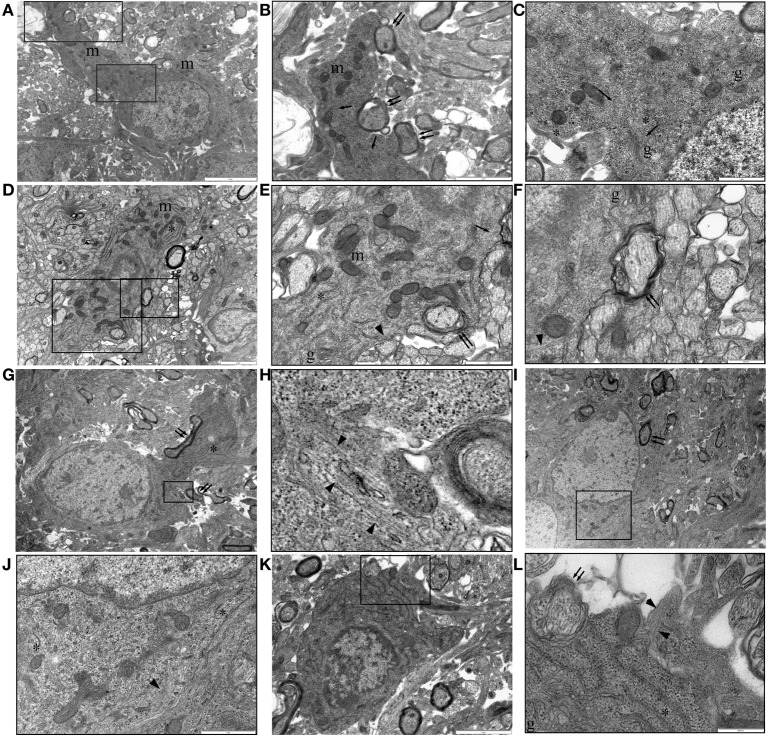
**P10 RON: Oligodendrocytes. (A–C)** Typical oligodendrocyte (boxed areas in “**A**” shown at higher gain in “**B**” and “**C**”). This cell contains numerous mitochondria (“m”), golgi-apparatus (“g”), narrow-bore ER (“^*^”), and microtubules (arrows). A large process is actively myelinating several large-diameter axons (double arrows). **(D–F)** An oligodendrocyte with an immature phenotype (boxed areas in “**C**” shown at higher gain in “**D**” and “**E**”). Mitochondria (“m”), golgi-apparatus (“g”), and narrow-bore ER (“^*^”) are present throughout the cytoplasm and several large diameter axons are in the early stages of myelination (double arrows). Microtubules (arrows) and glial filaments (arrow heads) are present in the cytoplasm. A large process is actively myelinating several large-diameter axons (double arrows). **(G,H)** An otherwise typical oligodendrocyte that is actively myelinated several axons (double arrows) contains a small area of glial filaments (arrow head). **(I,J)** Further example of a myelinating (double arrow) oligodendrocyte (boxed area shown at higher gain in “**J**”) containing glial filaments (arrow head). In both cases, note the typical narrow-bore ER (“^*^”) and nuclear morphology. **(K,L)** A less mature oligodendrocyte (note size and nuclear morphology) is starting to ensheath an axon (double arrows) and contains a glial filament bundle (arrow heads).

Oligodendrocytes can sometimes be unambiguously identified in P10 RON by the presence of myelinated sheaths contiguous with the cell membrane (Figure 7). The general form and distribution of organelles such as mitochondria and Golgi apparatus were not distinguishing features of these cells but nuclear morphology was generally spheroid and often contained a nucleolus. The chromatin was less likely to be clustered under the nuclear envelope than that found in astrocytes and a narrow-bore ER was a clear distinguishing feature (Figure 7). Oligodendrocytes in P10 nerves were a mixture of larger, more differentiated cells (Figures 7A–C) and smaller, less mature cells, which tended to have a more clustered chromatin and often lacked large processes (Figures 7D–F,K,L). This distinction has been made previously e.g., by (Vaughn and Peters, 1967; Vaughn, 1969; Skoff et al., 1976; Skoff, 1990) but it should be noted that these “immature” cells of the oligodendrocyte lineage sometimes had ensheathed neighboring axons (Figures 7E,L, double arrows) and may in fact be sections through cells at a level close to the nuclear envelope. Positively identified oligodendrocytes frequently expressed microtubules apparently randomly arrayed throughout the cytoplasm (Figures 7B,C,E, arrows) and in some cases contained loose bundles of glial filaments (Figures 7E,F) and regions of stacked glial filaments (Figures 7H,J,L).

**Figure 8:**
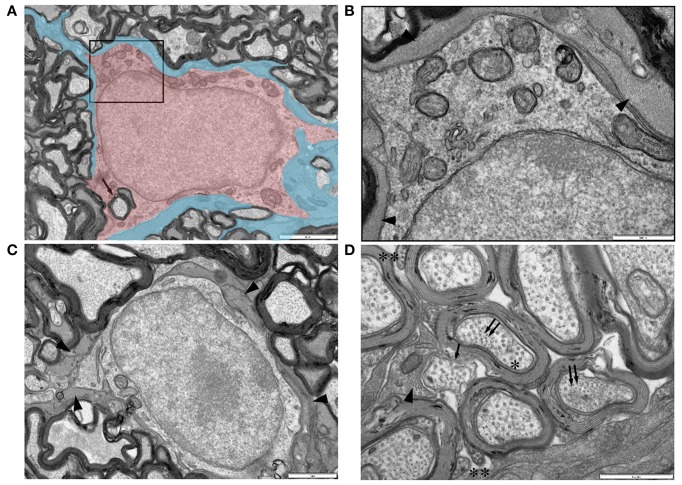
**Ultrastructural features of glia in adult RON. (A,B)** An oligodendrocyte (shaded red), identified on the basis that it completely encircles a myelinated axon in which the outer membrane is contiguous with the cell (arrow) (boxed area in “**A**” is shown at higher gain in “**B**”). Note the relatively uniform chromatin throughout the large nucleus and the numerous mitochondria in the soma. The cell is completely ensheathed by astrocyte processes, identified by the presence of glial filaments (**A**: shaded blue; **B**: arrow heads). **(C)** A further example of an astrocyte ensheathed oligodendrocyte, astrocyte processes indicated by arrow heads. **(D)** High-gain cross-section micrograph showing a non-myelinated section of an axon that may be a node of Ranvier (arrow). Note the close apposition of an astrocyte process which contains glial filaments in cross-section (arrow head) that are somewhat smaller than neuro-filaments in neighboring axons (double arrows). Axon microtubules (e.g., “^*^”) are indistinguishable from those in oligodendrocyte outer tongue processes on the outer spiral of myelin sheaths (“^**^”).

Oligodendrocytes are readily identifiable in adult RON due to their ensheathment of axons (e.g., Figure 8A, arrow) and have features previously described for this cell type. Glial filaments were not observed. A previously unremarked close morphological relationship between oligodendrocyte somata and astrocytes was often present, with oligodendrocytes completely encased in glial filament expressing astrocyte processes (Figures 8A–C, arrow heads). Astrocyte processes also made very close contact with the outer layer of the myelin sheath (Figure 8D) and with rare non-myelinated axon (Figure 8D).

## Discussion

NG-2(+) cells are OPCs capable of receiving synaptic input which may regulate their cell fate (Bergles et al., 2000, 2010; Kukley et al., 2007; Ziskin et al., 2007). These cells may also extend processes into the node of Ranvier and can transform into reactive astrocytes under pathological and cell culture conditions (Levine and Stallcup, 1987; Hirsch and Bahr, 1999; Leoni et al., 2009; Honsa et al., 2012). There have been several prior attempts to immuno-label NG2 protein for ultrastructural analysis of these cells, utilizing peroxides DAB pre-embedded approaches that do not preserve fine cellular characteristics (Levine and Card, 1987; Levine and Stallcup, 1987; Ong and Levine, 1999; Peters and Sethares, 2004; Leoni et al., 2009). Prior studies have noted that preservation of NG-2 reactivity requires light fixation and that NG-2(+) cells are particularly poorly preserved compared to other cell types (Peters and Sethares, 2004). This is consistent with our observation that staining is highly sensitive to fixation and embedding conditions. The current study is the first to describe the cellular features of these NG-2(+) cells, which constitute two distinct populations of cells in the P10 RON: astrocytes and OPCs.

### P10 RON astrocytes can express NG-2 and transiently ensheath axons

The results show that astrocytes in the developing RON identified by unambiguous ultrastructural features or by the commonly used GFAP(+) criterion widely express the NG-2 proteoglycan. We also observed co-expression in adult RON and in gray matter, with orthogonal immuno-fluorescent confocal image stacks indicating populations of GFAP/NG-2(+) cells in both regions. This observation is consistent with studies using GFP expression driven from the GFAP promoter, which reveal duel populations of GFP bright/NG-2(−) and GFP dim/NG-2(+) cells in several brain locations (Matthias et al., 2003; Grass et al., 2004; Leoni et al., 2009). Common recombination systems employing the GFAP promoter are inefficient and drive expression in only a small proportion of astrocytes, suggesting under reporting of NG-2 expression of cells with a GFAP(+) phenotype in these animals (Casper and McCarthy, 2006). There is also convincing evidence for NG-2/GFAP co-expression in astrocytes raised in culture conditions (e.g., Levine and Stallcup, 1987; Hirsch and Bahr, 1999), and for transformation of NG-2 expressing into GFAP expressing cells in organotypic slices (Leoni et al., 2009). Recent evidence implicates the Olig2 transcription factor in regulating NG-2 cell fate switching between the astrocyte and oligodendrocyte lineages (Zhu et al., 2012). NG-2 cells have been documented as GFAP(+) in a variety of forms of injury and disease models including in the area surrounding demyelination in multiple sclerosis (Nair et al., 2008) while viral demyelination evokes proliferation of O4(+)/GFAP(+) cells in the spinal cord (Godfraind et al., 1989), and anti-galactocerebroside (GC) induced demyelination resulted in proliferation of GFAP(+) GC(+) cells (Carroll et al., 1987). Numerous GFAP(+)/MBP(+) cells are reported in a mouse model of phenylketonuria, which is associated with central hypo-mylination (Dyer et al., 1996), and there is strong evidence for astrocyte production from NG-2 cells following traumatic injury (Carmen et al., 2007), ischemia (Honsa et al., 2012) and spinal cord injury (Wu et al., 2005). The picture is not straightforward however, since NG-2 transformation into GFAP(+) astrocytes does not appear to occur after neocortical stab injury (Komitova et al., 2011), while NG-2 expressing cells derived from optic nerve explants do not express GFAP (Merchan et al., 1965; Spassky et al., 2002).

While it is often stated that immuno-labeling studies show no NG2/GFAP co-expression in the CNS, examples where partial co-expression is apparent from published data include: (Redwine et al., 1997, Figure 2; Hamilton et al., 2009 Figure 4C; and Nishiyama et al., 1996 Figure 7C). Examples where NG2 and GFAP co-staining appears too complex to meaningfully distinguish include (Butt et al., 1999, Figure 2 and Polito and Reynolds, 2005, Figure 1A). The current data provide strong evidence that astrocyte-type functions ascribed to NG-2(+) cells are in fact examples of NG-2(+) astrocytes. For example, the extension of NG-2(+) glial processes into the node of Ranvier is based on pre-embedded DAB I-EM that fails to preserve sufficient ultrastructural detail to reveal the presence or absence of glial filaments (Leoni et al., 2009). The presence of GFAP(+) / NG-2(+) astrocytes in the nerve and the well-documented extension of astrocyte processes into the node of Ranvier suggest that these NG-2(+) processes are astrocytic in nature.

In P0 and P10 RON, astrocyte finger processes were observed to extend around axons, on occasion depositing several layers of membrane. These processes differed from those extended by oligodendroglia, in that they frequently contained glial filaments, did not appear to discriminate in terms of axon diameter, and never produced compact myelin. Axon encirclement by astrocyte processes was not observed in adult RON or at nodes of Ranvier and must therefore be a transient phenomenon. Astrocyte finger processes have been described before (Vaughn and Peters, 1967; Lord and Duncan, 1987; Wolff, 2007), but axon wrapping of several membrane layers has not been recognized. The functional significance is not clear and there were no specific cellular inclusions within either axons or glial processes at these specializations.

### Immature oligodendroglia can retain glial filaments

In addition to NG-2 expression in astrocytes in neonatal white matter, a number of immature oligodendrocytes were found to contain glial filaments. Considering the GFAP(+) stem cell origin of many oligodendrocytes (Malatesta et al., 2003; Casper and McCarthy, 2006), and the exceptional stability of filamentous GFAP [half-life ~8 days (Chiu and Goldman, 1984)], the presence of a low number of glial filaments is predicted in immature oligodendroglia. There are prior reports of GFAP expression in cells of the early oligodendroglial lineage. For example, GFAP(+)/MBP(+) cells have been described in fetal human and mouse spinal white matter (Choi and Kim, 1984, 1985; Choi, 1986) and GFAP(+)/myelin oligodendrocyte-specific protein (MOSP)(+) cells in central white matter tracts (Dyer et al., 2000). Clonal analysis indicates a shared cell fate for some astrocytes and oligodendrocytes in the brain (Levison and Goldman, 1993; Zerlin et al., 2004), while OPCs transplanted into glial depleted CNS generate both oligodendrocytes and astrocytes (Franklin et al., 1995; Windrem et al., 2004). However, the current observation of glial filament-rich astrocyte encapsulation of mature oligodendrocytes will make the spatial resolution of these two glial elements difficult. Indeed, the coexistence of GFAP(+) / NG-2(+) and GFAP(+) / NG-2(−)astrocytes, the presence of glial filaments in immature oligodendroglia, the multiple layer wrapping of immature axons by astrocytes, and the encapsulation of mature oligodendrocytes by astrocytes are all phenomena that make the identification and differentiation of the two cell types problematic.

### Transgenic studies of oligodendroglial origins

Studies using transgenic reporter targeting to glial specific promoters paint a confused picture, with several lines of evidence suggesting that some astrocytes share a lineage with oligodendrocytes. NG-2 (Zhu et al., 2008; Komitova et al., 2009), PLP (Guo et al., 2010; Michalski et al., 2011), and PLP/Olig2 (Chung et al., 2013) promoters report GFAP immuno-reactive progeny variously in ventral brain, olfactory bulb, spinal cord, optic nerve, and cerebellum. The Olig2 promoter shows reporter expression in GFAP(+) cells throughout the brain (Dimou et al., 2008), and MBP-lacZ mice show a similar pattern of reporter/GFAP co-expression (Dyer et al., 2000). In addition to these reports of GFAP(+) progeny in oligodendroglial-specific promoter lineage cells, NG-2(+) cells have been reported in the developing spinal cord that are negative for reporter in oligodendrocyte lineage promoters such as CNP-GFP (Yuan et al., 2002; Lytle et al., 2009). (Lytle et al., 2009) suggest that these cells are immature astrocytes because some express the astrocyte marker S-100β. Similar results are reported in reporter expressing cells throughout the brain at all post-natal points by Karram et al. (2008) using an NG2 knock in.

Contradicting these reports suggesting a shared origin for some populations of astrocytes and oligodendrocytes, PDGFαR-CreER mice show no astrocyte progeny (Rivers et al., 2008; Kang et al., 2010). PDGFαR(+) cells are also consistently found to be NG-2(+) (Nishiyama et al., 1996; Rivers et al., 2008; He et al., 2009). However, the reverse is not the case and the ratio of NG-2(+) cells that express reporter in PDGFαR-GFP mice varies between ~30–90%, depending on location and post-natal age (Clarke et al., 2012). There are also reports of NG-2(+) cells that are PDGFαR(−) at particular points in development (Diers-Fenger et al., 2001; Liu et al., 2002; Wilson et al., 2006; He et al., 2009), although these data contrast with the work of (Rivers et al., 2008) and (Kang et al., 2010), who found 100% co-expression of both markers in the adult mouse.

The current findings of NG-2 expression in astrocytes identified by ultrastructural and immuno-label approaches confirm the non-selective nature of NG-2 expression with, as a minimum, some populations of astrocytes expressing the antigen (see Richardson et al., 2011). This observation and the data showing the presence of glial filaments in immature oligodendroglia, which may contribute to the prior reports of GFAP(+) cells in transgenic mice with oligodendroglia-specific reporter expression, emphasize the difficulty of positively identifying either astrocytes or oligodendrocytes via transgenic or immuno-labeling approaches alone.

### Conflict of interest statement

The authors declare that the research was conducted in the absence of any commercial or financial relationships that could be construed as a potential conflict of interest.

## References

[B1] Alix J. J. Dolphin A. C. Fern R. (2008). Vesicular apparatus, including functional calcium channels, are present in developing rodent optic nerve axons and are required for normal node of Ranvier formation. J. Physiol. 586(Pt 17), 4069–4089. 10.1113/jphysiol.2008.155077 18599536 PMC2652192

[B2] Alix J. J. Fern R. (2009). Glutamate receptor-mediated ischemic injury of premyelinated central axons. Ann. Neurol. 66, 682–693. 10.1002/ana.21767 19938170

[B3] Arranz A. M. Hussein A. Alix J. J. Perez-Cerda F. Allcock N. Matute C.. (2008). Functional glutamate transport in rodent optic nerve axons and glia. Glia 56, 1353–1367. 10.1002/glia.20703 18551624

[B4] Barres B. A. Hart I. K. Coles H. S. Burne J. F. Voyvodic J. T. Richardson W. D.. (1992). Cell death and control of cell survival in the oligodendrocyte lineage. Cell 70, 31–46. 10.1016/0092-8674(92)90531-G 1623522

[B5] Bergles D. E. Jabs R. Steinhauser C. (2010). Neuron-glia synapses in the brain. Brain Res. Rev. 63, 130–137. 10.1016/j.brainresrev.2009.12.003 20018210 PMC2862892

[B6] Bergles D. E. Roberts J. D. Somogyi P. Jahr C. E. (2000). Glutamatergic synapses on oligodendrocyte precursor cells in the hippocampus. Nature 405, 187–191. 10.1038/35012083 10821275

[B6a] Butt A. M. Duncan A. Hornby M. F. Kirvell S. L. Hunter A. Levine J. M.. (1999). Cells expressing the NG2 antigen contact nodes of Ranvier in adult CNS white matter. Glia 26, 84–91. 10088675

[B7] Carmen J. Magnus T. Cassiani-Ingoni R. Sherman L. Rao M. S. Mattson M. P. (2007). Revisiting the astrocyte-oligodendrocyte relationship in the adult CNS. Prog. Neurobiol. 82, 151–162. 10.1016/j.pneurobio.2007.03.001 17448587

[B8] Carroll W. M. Jennings A. R. Mastaglia F. L. (1987). Reactive glial cells in CNS demyelination contain both GC and GFAP. Brain Res. 411, 364–369. 3300847 10.1016/0006-8993(87)91088-2

[B9] Casper K. B. McCarthy K. D. (2006). GFAP-positive progenitor cells produce neurons and oligodendrocytes throughout the CNS. Mol. Cell. Neurosci. 31, 676–684. 10.1016/j.mcn.2005.12.006 16458536

[B10] Chiu F. C. Goldman J. E. (1984). Synthesis and turnover of cytoskeletal proteins in cultured astrocytes. J. Neurochem. 42, 166–174. 10.1111/j.1471-4159.1984.tb09713.x 6689687

[B11] Choi B. H. (1986). Glial fibrillary acidic protein in radial glia of early human-fetal cerebrum—a light and electron-microscopic immunoperoxidase study. J. Neuropathol. Exp. Neurol. 45, 408–418. 10.1097/00005072-198607000-00003 3522808

[B12] Choi B. H. Kim R. C. (1984). Expression of glial fibrillary acidic protein in immature oligodendroglia. Science 223, 407–409. 10.1126/science.6197755 6197755

[B13] Choi B. H. Kim R. C. (1985). Expression of glial fibrillary acidic protein by immature oligodendroglia and its implications. J. Neuroimmunol. 8, 215–235. 10.1016/S0165-5728(85)80064-3 2409106

[B14] Chung S. H. Guo F. Jiang P. Pleasure D. E. Deng W. (2013). Olig2/Plp-positive progenitor cells give rise to Bergmann glia in the cerebellum. Cell Death Dis. 4:e546. 10.1038/cddis.2013.74 23492777 PMC3615735

[B15] Clarke L. E. Young K. M. Hamilton N. B. Li H. Richardson W. D. Attwell D. (2012). Properties and fate of oligodendrocyte progenitor cells in the corpus callosum, motor cortex, and piriform cortex of the mouse. J. Neurosci. 32, 8173–8185. 10.1523/JNEUROSCI.0928-12.2012 22699898 PMC3378033

[B16] Diers-Fenger M. Kirchhoff F. Kettenmann H. Levine J. M. Trotter J. (2001). AN2/NG2 protein-expressing glial progenitor cells in the murine CNS: isolation, differentiation, and association with radial glia. Glia 34, 213–228. 10.1002/glia.1055 11329183

[B17] Dimou L. Simon C. Kirchhoff F. Takebayashi H. Gotz M. (2008). Progeny of Olig2-expressing progenitors in the gray and white matter of the adult mouse cerebral cortex. J. Neurosci. 28, 10434–10442. 10.1523/JNEUROSCI.2831-08.2008 18842903 PMC6671038

[B18] Dyer C. A. Kendler A. Jean-Guillaume D. Awatramani R. Lee A. Mason L. M.. (2000). GFAP-positive and myelin marker-positive glia in normal and pathologic environments. J. Neurosci. Res. 60, 412–426. 10.1002/(SICI)1097-4547(20000501)60:3<412::AID-JNR16>3.3.CO;2-5 10797544

[B19] Dyer C. A. Kendler A. Philibotte T. Gardiner P. Cruz J. Levy H. L. (1996). Evidence for central nervous system glial cell plasticity in phenylketonuria. J. Neuropathol. Exp. Neurol. 55, 795–814. 10.1097/00005072-199607000-00005 8965095

[B20] Federoff S. Vernadakis A. (1986). Astrocytes Pt 1: Development, Morphology, and Regional Specialization of Astrocytes. London: Academicd Press Inc.

[B21] Franklin R. J. Bayley S. A. Milner R. Ffrench-Constant C. Blakemore W. F. (1995). Differentiation of the O-2A progenitor cell line CG-4 into oligodendrocytes and astrocytes following transplantation into glia-deficient areas of CNS white matter. Glia 13, 39–44. 10.1002/glia.440130105 7751054

[B22] Godfraind C. Friedrich V. L. Holmes K. V. Dubois-Dalcq M. (1989). Dubois-Dalcq: *in vivo* analysis of glial cell phenotypes during a viral demyelinating disease in mice. J. Cell Biol. 109, 2405–2416. 10.1083/jcb.109.5.2405 2553746 PMC2115831

[B23] Grass D. Pawlowski P. G. Hirrlinger J. Papadopoulos N. Richter D. W. Kirchhoff F.. (2004). Diversity of functional astroglial properties in the respiratory network. J. Neurosci. 24, 1358–1365. 10.1523/JNEUROSCI.4022-03.2004 14960607 PMC6730324

[B24] Greenwood K. Butt A. M. (2003). Evidence that perinatal and adult NG2-glia are not conventional oligodendrocyte progenitors and do not depend on axons for their survival. Mol. Cell. Neurosci. 23, 544–558. 10.1016/S1044-7431(03)00176-3 12932436

[B25] Guo F. Maeda Y. Ma J. Xu J. Horiuchi M. Miers L.. (2010). Pyramidal neurons are generated from oligodendroglial progenitor cells in adult piriform cortex. J. Neurosci. 30, 12036–12049. 10.1523/JNEUROSCI.1360-10.2010 20826667 PMC2940828

[B25a] Hamilton N. Hubbard P. S. Butt A. M. (2009). Effects of glutamate receptor activation on NG2-glia in the rat optic nerve. J. Anat. 214, 208–218. 10.1111/j.1469-7580.2008.01017.x 19207982 PMC2667878

[B26] He Y. Cai W. Wang L. Chen P. (2009). A developmental study on the expression of PDGFalphaR immunoreactive cells in the brain of postnatal rats. Neurosci. Res. 65, 272–279. 10.1016/j.neures.2009.07.011 19665498

[B27] Hirsch S. Bahr M. (1999). Immunocytochemical characterization of reactive optic nerve astrocytes and meningeal cells. Glia 26, 36–46. 10088670 10.1002/(sici)1098-1136(199903)26:1<36::aid-glia4>3.0.co;2-c

[B28] Honsa P. Pivonkova H. Dzamba D. Filipova M. Anderova M. (2012). Polydendrocytes display large lineage plasticity following focal cerebral ischemia. PLoS ONE 7:e36816. 10.1371/journal.pone.0036816 22590616 PMC3349640

[B29] Kang S. H. Fukaya M. Yang J. K. Rothstein J. D. Bergles D. E. (2010). NG2+ CNS glial progenitors remain committed to the oligodendrocyte lineage in postnatal life and following neurodegeneration. Neuron 68, 668–681. 10.1016/j.neuron.2010.09.009 21092857 PMC2989827

[B30] Karram K. Goebbels S. Schwab M. Jennissen K. Seifert G. Steinhauser C.. (2008). NG2-expressing cells in the nervous system revealed by the NG2-EYFP-knockin mouse. Genesis 46, 743–757. 10.1002/dvg.20440 18924152

[B31] Komitova M. Serwanski D. R. Lu Q. R. Nishiyama A. (2011). NG2 cells are not a major source of reactive astrocytes after neocortical stab wound injury. Glia 59, 800–809. 10.1002/glia.21152 21351161 PMC3560299

[B32] Komitova M. Zhu X. Serwanski D. R. Nishiyama A. (2009). NG2 cells are distinct from neurogenic cells in the postnatal mouse subventricular zone. J. Comp. Neurol. 512, 702–716. 10.1002/cne.21917 19058188 PMC2614367

[B33] Kukley M. Capetillo-Zarate E. Dietrich D. (2007). Vesicular glutamate release from axons in white matter. Nat. Neurosci. 10, 311–320. 10.1038/nn1850 17293860

[B34] Leoni G. Rattray M. Butt A. M. (2009). NG2 cells differentiate into astrocytes in cerebellar slices. Mol. Cell. Neurosci. 42, 208–218. 10.1016/j.mcn.2009.07.007 19616628

[B35] Levine J. M. Card J. P. (1987). Light and electron microscopic localization of a cell surface antigen (NG2) in the rat cerebellum: association with smooth protoplasmic astrocytes. J. Neurosci. 7, 2711–2720. 3305798 10.1523/JNEUROSCI.07-09-02711.1987 PMC6569120

[B36] Levine J. M. Stallcup W. B. (1987). Plasticity of developing cerebellar cells *in vitro* studied with antibodies against the NG2 antigen. J. Neurosci. 7, 2721–2731. 3305799 10.1523/JNEUROSCI.07-09-02721.1987 PMC6569134

[B37] Levison S. W. Goldman J. E. (1993). Both oligodendrocytes and astrocytes develop from progenitors in the subventricular zone of postnatal rat forebrain. Neuron 10, 201–212. 10.1016/0896-6273(93)90311-E 8439409

[B38] Liu L. Korzh V. Balasubramaniyan N. V. Ekker M. Ge R. (2002). Platelet-derived growth factor A (pdgf-a) expression during zebrafish embryonic development. Dev. Genes Evol. 212, 298–301. 10.1007/s00427-002-0234-3 12211169

[B39] Lord K. E. Duncan I. D. (1987). Early postnatal development of glial cells in the canine cervical spinal cord. J. Comp. Neurol. 265, 34–46. 10.1002/cne.902650104 3693603

[B40] Lytle J. M. Chittajallu R. Wrathall J. R. Gallo V. (2009). NG2 cell response in the CNP-EGFP mouse after contusive spinal cord injury. Glia 57, 270–285. 10.1002/glia.20755 18756526 PMC2696059

[B41] Malatesta P. Hack M. A. Hartfuss E. Kettenmann H. Klinkert W. Kirchhoff F.. (2003). Neuronal or glial progeny: regional differences in radial glia fate. Neuron 37, 751–764. 10.1016/S0896-6273(03)00116-8 12628166

[B42] Matthias K. Kirchhoff F. Seifert G. Huttmann K. Matyash M. Kettenmann H.. (2003). Segregated expression of AMPA-type glutamate receptors and glutamate transporters defines distinct astrocyte populations in the mouse hippocampus. J. Neurosci. 23, 1750–1758. 12629179 10.1523/JNEUROSCI.23-05-01750.2003 PMC6741945

[B43] Merchan P. Bribian A. Sanchez-Camacho C. Lezameta M. Bovolenta P. de Castro F. (1965). Sonic hedgehog promotes the migration and proliferation of optic nerve oligodendrocyte precursors. Mol. Cell. Neurosci. 36, 355–368. 10.1016/j.mcn.2007.07.012 17826177

[B44] Michalski J. P. Anderson C. Beauvais A. De Repentigny Y. Kothary R. (2011). The proteolipid protein promoter drives expression outside of the oligodendrocyte lineage during embryonic and early postnatal development. PLoS ONE 6:e19772 10.1371/journal.pone.0019772 21572962 PMC3091881

[B45] Nair A. Frederick T. J. Miller S. D. (2008). Astrocytes in multiple sclerosis: a product of their environment. Cell. Mol. Life Sci. 65, 2702–2720. 10.1007/s00018-008-8059-5 18516496 PMC2858316

[B46] Nishiyama A. Komitova M. Suzuki R. Zhu X. (2009). Polydendrocytes (NG2 cells): multifunctional cells with lineage plasticity. Nat. Rev. Neurosci. 10, 9–22. 10.1038/nrn2495 19096367

[B47] Nishiyama A. Lin X. H. Giese N. Heldin C. H. Stallcup W. B. (1996). Co-localization of NG2 proteoglycan and PDGF alpha-receptor on O2A progenitor cells in the developing rat brain. J. Neurosci. Res. 43, 299–314. 8714519 10.1002/(SICI)1097-4547(19960201)43:3<299::AID-JNR5>3.0.CO;2-E

[B48] Ong W. Y. Levine J. M. (1999). A light and electron microscopic study of NG2 chondroitin sulfate proteoglycan-positive oligodendrocyte precursor cells in the normal and kainate-lesioned rat hippocampus. Neuroscience 92, 83–95. 10.1016/S0306-4522(98)00751-9 10392832

[B49] Peters A. Sethares C. (2004). Oligodendrocytes, their progenitors and other neuroglial cells in the aging primate cerebral cortex. Cereb. Cortex 14, 995–1007. 10.1093/cercor/bhh060 15115733

[B49a] Polito A. Reynolds R. (2005). NG2-expressing cells as oligodendrocyte progenitors in the normal and demyelinated adult central nervous system. J. Anat. 207, 707–716. 10.1111/j.1469-7580.2005.00454.x 16367798 PMC1571577

[B50] Raff M. C. Abney E. R. Cohen J. Lindsay R. Noble M. (1983). Two types of astrocytes in cultures of developing rat white matter: differences in morphology, surface gangliosides, and growth characteristics. J. Neurosci. 3, 1289–1300. 6343560 10.1523/JNEUROSCI.03-06-01289.1983 PMC6564607

[B50a] Redwine J. M. Blinder K. L. Armstrong R. C. (1997). *In situ* expression of fibroblast growth factor receptors by oligodendrocyte progenitors and oligodendrocytes in adult mouse central nervous system. J. Neurosci. Res. 50, 229–237. 9373032 10.1002/(SICI)1097-4547(19971015)50:2<229::AID-JNR11>3.0.CO;2-3

[B51] Reyners H. Gianfelici de Reyners E. Maisin J. R. (1982). The beta astrocyte: a newly recognized radiosensitive glial cell type in the cerebral cortex. J. Neurocytol. 11, 967–983. 10.1007/BF01148311 7153792

[B52] Richardson W. D. Young K. M. Tripathi R. B. McKenzie I. (2011). NG2-glia as multipotent neural stem cells: fact or fantasy? Neuron 70, 661–673. 10.1016/j.neuron.2011.05.013 21609823 PMC3119948

[B53] Rivers L. E. Young K. M. Rizzi M. Jamen F. Psachoulia K. Wade A.. (2008). PDGFRA/NG2 glia generate myelinating oligodendrocytes and piriform projection neurons in adult mice. Nat. Neurosci. 11, 1392–1401. 10.1038/nn.2220 18849983 PMC3842596

[B54] Skoff R. P. (1990). Gliogenesis in rat optic nerve: astrocytes are generated in a single wave before oligodendrocytes. Dev. Biol. 139, 149–168. 10.1016/0012-1606(90)90285-Q 2328833

[B55] Skoff R. P. Price D. L. Stocks A. (1976). Electron microscopic autoradiographic studies of gliogenesis in rat optic nerve. I. Cell proliferation. J. Comp. Neurol. 169, 291–312. 10.1002/cne.901690303 972201

[B56] Small R. K. Riddle P. Noble M. (1987). Evidence for migration of oligodendrocyte—type-2 astrocyte progenitor cells into the developing rat optic nerve. Nature 328, 155–157. 10.1038/328155a0 3600791

[B57] Spassky N. de Castro F. Le Bras B. Heydon K. Queraud-LeSaux F. Bloch-Gallego E.. (2002). Directional guidance of oligodendroglial migration by class 3 semaphorins and netrin-1. J. Neurosci. 22, 5992–6004. 12122061 10.1523/JNEUROSCI.22-14-05992.2002 PMC6757938

[B58] Vaughn J. E. (1969). An electron microscopic analysis of gliogenesis in rat optic nerves. Z. Zellforsch. Mikrosk. Anat. 94, 293–324. 10.1007/BF00319179 4893051

[B59] Vaughn J. E. Peters A. (1967). Electron microscopy of the early postnatal development of fibrous astrocytes. Am. J. Anat. 121, 131–152. 10.1002/aja.1001210109 4861133

[B60] Vaughn J. E. Peters A. (1968). A third neuroglial cell type. An electron microscopic study. J. Comp. Neurol. 133, 269–288. 10.1002/cne.901330207 4878436

[B61] Wigley R. Butt A. M. (2009). Integration of NG2-glia (synantocytes) into the neuroglial network. Neuron Glia Biol. 5, 21–28. 10.1017/S1740925X09990329 19785922

[B62] Wilson H. C. Scolding N. J. Raine C. S. (2006). Co-expression of PDGF alpha receptor and NG2 by oligodendrocyte precursors in human CNS and multiple sclerosis lesions. J. Neuroimmunol. 176, 162–173. 10.1016/j.jneuroim.2006.04.014 16753227

[B63] Windrem M. S. Nunes M. C. Rashbaum W. K. Schwartz T. H. Goodman R. A. McKhann G. II.. (2004). Fetal and adult human oligodendrocyte progenitor cell isolates myelinate the congenitally dysmyelinated brain. Nat. Med. 10, 93–97. 10.1038/nm974 14702638

[B64] Wolff J. (2007). [Electron microscopic investigations of the structure and form of astrocyte porcesses] (Ger)processes]. Z. Zellforsch. Mikrosk. Anat. 66, 811–828. 10.1007/BF00342958 5857787

[B65] Wu D. Shibuya S. Miyamoto O. Itano T. Yamamoto T. (2005). Increase of NG2-positive cells associated with radial glia following traumatic spinal cord injury in adult rats. J. Neurocytol. 34, 459–469. 10.1007/s11068-006-8998-4 16902766

[B66] Yuan X. Chittajallu R. Belachew S. Anderson S. McBain C. J. Gallo V. (2002). Expression of the green fluorescent protein in the oligodendrocyte lineage: a transgenic mouse for developmental and physiological studies. J. Neurosci. Res. 70, 529–545. 10.1002/jnr.10368 12404507

[B67] Zerlin M. Milosevic A. Goldman J. E. (2004). Glial progenitors of the neonatal subventricular zone differentiate asynchronously, leading to spatial dispersion of glial clones and to the persistence of immature glia in the adult mammalian CNS. Dev. Biol. 270, 200–213. 10.1016/j.ydbio.2004.02.024 15136150

[B68] Zhu M. Chen M. Lichtler A. C. O'Keefe R. J. Chen D. (2008). Tamoxifen-inducible Cre-recombination in articular chondrocytes of adult Col2a1-CreER(T2) transgenic mice. Osteoarthritis Cartilage 16, 129–130. 10.1016/j.joca.2007.08.001 17888690 PMC2271067

[B69] Zhu X. Zuo H. Maher B. J. Serwanski D. R. LoTurco J. J. Lu Q. R.. (2012). Olig2-dependent developmental fate switch of NG2 cells. Development 139, 2299–2307. 10.1242/dev.078873 22627280 PMC3367441

[B70] Ziskin J. L. Nishiyama A. Rubio M. Fukaya M. Bergles D. E. (2007). Vesicular release of glutamate from unmyelinated axons in white matter. Nat. Neurosci. 10, 321–330. 10.1038/nn1854 17293857 PMC2140234

